# A multi-nutrient supplement reduced markers of inflammation and improved physical performance in active individuals of middle to older age: a randomized, double-blind, placebo-controlled study

**DOI:** 10.1186/1475-2891-10-90

**Published:** 2011-09-07

**Authors:** Courtenay Dunn-Lewis, William J Kraemer, Brian R Kupchak, Neil A Kelly, Brent A Creighton, Hui-Ying Luk, Kevin D Ballard, Brett A Comstock, Tunde K Szivak, David R Hooper, Craig R Denegar, Jeff S Volek

**Affiliations:** 1 Human Performance Laboratory, Department of Kinesiology, University of Connecticut, Storrs, CT, 06269, USA

**Keywords:** Aging, Supplementation, Resistance Exercise, Amino Acids, Joint, Recovery, Vitamin B

## Abstract

**Background:**

While exercise acts to combat inflammation and aging, the ability to exercise may itself be compromised by inflammation and inflammation's impact on muscle recovery and joint inflammation. A number of nutritional supplements have been shown to reduce inflammation and improve recovery. The purpose of the current investigation was to examine the effect of a multi-nutrient supplement containing branched chain amino acids, taurine, anti-inflammatory plant extracts, and B vitamins on inflammatory status, endothelial function, physical function, and mood in middle-aged individuals.

**Methods:**

Thirty-one healthy and active men (N = 16, mean age 56 ± 6.0 yrs) and women (N = 15, mean age = 52 ± 7.5 yrs) participated in this investigation. Subjects completed one 28 day cycle of placebo supplementation and one 28 day cycle of multi-nutrient supplementation (separated by a one week washout period) in a balanced, randomized, double-blind, cross-over design. Subjects completed weekly perceptual logs (PROMIS-57, KOOS) and pre- and post- testing around the supplementation period. Testing consisted of brachial artery flow mediated dilation (FMD), blood measures, and physical performance on vertical jump, handgrip strength, and balance (dispersion from center of pressure). Significance for the investigation was p ≤ 0.05.

**Results:**

IL-6 significantly decreased in both men (from 1.2 ± 0.2 to 0.7 ± 0.4 pg·mL^-1^) and women (from 1.16 ± 0.04 to 0.7 ± 0.4 pg·mL^-1^). Perceived energy also improved for both men (placebo: 1.8 ± 0.7; supplement: 3.7 ± 0.8 AUC) and women (placebo: 1.2 ± 0.7; supplement: 2.8 ± 0.8 AUC). Alpha-1-antichymotrypsin (from 108.9 ± 38.6 to 55.5 ± 22.2 ug·mL^-1^), Creatine Kinase (from 96 ± 34 to 67 ± 23 IU·L^-1^), general pain, and joint pain decreased in men only, while anxiety and balance (from 0.52 ± 0.13 to 0.45 ± 0.12 cm) improved in women only. Men showed increased performance in vertical jump power (from 2642 ± 244 to 3134 ± 282 W) and grip strength (from 42.1 ± 5.9 to 48.5 ± 4.9 kg).

**Conclusions:**

A multi-nutrient supplement is effective in improving inflammatory status in both men and women, markers of pain, joint pain, strength, and power in men only, and both anxiety and balance (a risk factor for hip fracture) in women. Therefore, a multi-nutrient supplement may help middle-aged individuals to prolong physical function and maintain a healthy, active lifestyle.

## Background

Aging has long been portrayed as an unpredictable, indiscriminate process of degeneration in cognitive and physical function. This harsh view has been challenged by our modern understanding of the role that age-related increases in inflammation play in chronic disease and aging [1]. It now appears that physiological well-being at advanced age may be influenced by the everyday anti-inflammatory habits of the individual [2]. Simple lifestyle choices, such as physical exercise and dietary patterns, may address chronic systemic inflammation and improve long-term health and function [3,4]. We now appreciate that inflammatory cytokines may be influenced by habitual behavior; this includes Interleukin-6 (IL-6), an inflammatory cytokine strongly associated with cognitive impairment [5], functional decline, loss of strength, sarcopenia, and mortality [6]. Indeed, exercise and nutrition may attenuate even the processes of aging that were once considered inevitable, such as declines in cognitive function, mood, sarcopenia, quality of life [7]. Our modern concept therefore suggests that although the processes of aging and inflammation certainly cannot be halted, it may be possible to extend cognitive and physical function long-term through appropriate diet and exercise practices.

While exercise acts as an important anti-inflammatory aid, the ability to recover from exercise in and of itself may be subject to the challenges of age-related inflammation. Aging is accompanied by increased free radical formation and circulatory changes that exacerbate inflammatory processes. This inflammatory cascade may include an increase in IL-6 - ordinarily an important component of muscle hypertrophy, but an inhibitor of muscle recovery at elevated concentrations [8]. IL-6 promotes chronic inflammation [9] and plays a large role in joint inflammation [10,11]. IL-6 contributes to the production of ACT [12] and together, the two inflammatory markers are associated with a two- to three-fold risk of reduced muscle strength in older adults [13]. At the same time, upstream hormonal signal processes, which are vital in gene expression and protein synthesis for both contractile and non-contractile proteins in skeletal muscle, also decline with age. The recovery of muscle and tissue slows with age, repair and remodeling of muscle is compromised, and inflammatory joint pain may persist (even in the absence of rheumatoid arthritis). Osteoporosis becomes a increasing concern, particularly in women; with age, the incidence of falling and the severity of fall-related injury increases dramatically, resulting in increased nursing home admissions [14] and hip fractures that significantly increase risk of mortality [15]. Furthermore, vascular endothelial dysfunction (as demonstrated by reduced brachial artery flow mediated dilation (FMD)) has been reported in healthy, sedentary middle-aged and older adults [16,17], and is regarded as an early manifestation of CVD [18]. Brachial artery FMD is inversely related to plasma markers of inflammation [19] and inflammation-induced oxidative stress potentially mediates endothelial dysfunction [20] in middle-aged and older adults (thus interventions that improve endothelial homeostasis may have important clinical implications to reduce CVD-related morbidity and mortality). Combined, these factors result in progressive functional limitations and disability. As such, while exercise is an important factor in fighting inflammation, aging individuals may need additional nutritional support to combat the effect of inflammation on the body, improve recovery from and adaptation to exercise, and therefore permit them to remain physically active with age.

Given the importance of chronic inflammation and recovery to health, a number of studies have found that certain nutritional supplements, in combination with a healthy, balanced diet and exercise, may allow for improved recovery and anti-inflammatory action [21]. Branched chain amino acids (BCAA), for example, have analgesic properties [22] and may inhibit the breakdown of muscle protein and enhance muscle protein synthesis [23]; this has been particularly shown with leucine supplementation [24]. Taurine, also amino acid, acts as a powerful anti-inflammatory that may reduce IL-6 production [25]. Green tea, a source of polyphenols, has chelating [26], anti-inflammatory, antibacterial, anti-mutagenic, anti-diabetic, and hypocholesterolemic properties [27]. It may help in cancer prevention, weight loss or management, stroke prevention, and prevention of cardiovascular disease [28]. Quercetin, another anti-inflammatory aid [29], may inhibit mast cell activation [30], serve as a psychostimulant, and improve physiological performance, health, and disease resistance [31]. Cat's claw or *uncaria tomentosa*, a wood-like vine, is another powerful antioxidant and anti-inflammatory [32]. In a separate role, B-vitamins have limited anti-inflammatory properties but are cofactors, crucial in metabolism, may increase energy (especially if diet is poor), and may improve mood and stress [33,34]. Therefore, a number of substances exist that improve age-related recovery and inflammation in distinct and independent ways.

While each of the nutritional substances noted impart powerful physiological effects, each act independently and individually lacks the benefits that others may possess. Thus, the multifaceted impact of inflammation and aging on exercise performance may benefit from a multidimensional supplement that includes each of these substances in a single supplement. Although the ingredients independently have been shown to contribute to amino acid signaling, reduction of inflammation, and recovery, it is not currently known if a combined supplement would benefit recovery, joint health, remodeling of muscle, and energy in healthy individuals attempting to remain physically active. Therefore, the purpose of this study is to examine the effects of a multi-nutrient supplement on physical performance, endothelial function, fatigue, mood, and other recovery factors in active men and women from the ages 40-70 years.

## Methods

### Experimental Design

**Figure 1:**
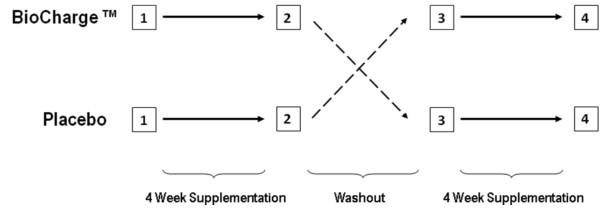
**Experimental Design**. Participants were randomly assigned to begin with either the multi-nutrient supplement or placebo supplement to begin in a balanced fashion. After a wash-out period, subjects completed the opposite supplement. Testing occurred immediately prior to and immediately following the 28-day supplementation periods.

This investigation utilized a balanced, cross-over, within-group, placebo-controlled design to examine the effects of the multi-nutrient supplement (*BioCharge*^®^, Advocare, Plano, TX; please see Table 1 for ingredients) on physical performance, joint health, fatigue, mood, and inflammation during the recovery period of active subjects. (The placebo included the same natural raspberry flavor, sucralose, and natural beet-derived color that matched the natural form and color of the active supplement). As seen in Figure 1, the study consisted of two cycles (one cycle of *BioCharge*^®^, one of placebo, the order randomly assigned in a balanced fashion). The funding agency blinded the supplement and placebo (as A and B). No indication of this assignment was provided (either on the packaging or otherwise) to any member of the study staff or any subject until after data collection and analysis had ceased. Each cycle required four weeks (28 days) of supplementation between its first and last visit at which time subjects mixed one packet of the powdered supplement or an identical but otherwise inactive placebo each day with 4-8 ounces of water. Between the two cycles there was a washout period of at least one week and subjects were asked to maintain the same level of activity between both cycles (as verified by activity questionnaire). The four testing visits occurred immediately before and immediately following the supplementation period. Testing consisted of assessment of endothelial function via high-resolution ultrasonography, blood draws, and physical tests (handgrip, quiet standing, and vertical jumps). In addition, at the end of each week of each supplementation cycle, subjects filled out a logbook at home. Registered dieticians ensured compliance to supplement protocol and daily consumption.

**Table 1:** Ingredients of Biocharge^® ^supplement

Supplement Facts Serving Size: 1 packet added to water Servings Per Container: 14 packets per carton		
	**Amt Per** **Serving**	**%DV**

**B Vitamins**		

Vitamin B12	0.25 mg	4200%

Vitamin B6	6 mg	300%

Folic acid (Vitamin B9)	0.40 mg	100%

Pantothenic Acid (Vitamin B5)	20 mg	200%

**Amino Acids**		

Taurine	500 mg	*

1-Leucine	2000 mg	*

Isoleucine	500 mg	*

Valine	500 mg	*

**Plant Extracts**		

Cat's Claw (uncaria tomentosa)	100 mg	*

Quercitin	100 mg	*

Green tea	50 mg	*

Biovin grape extract (full spectrum whole grape extract). Contains Proanthocyanidins (OPC), Polyphenols, and Trans-resverattrol	25 mg	*

*Daily Value (DV) not established		

Other ingredients: natural and artificial flavor, citric acid, sucralose. The supplement is comprised of food-grade ingredients. It contains ingredients that the FDA has either approved as food additives or considers "generally recognized as safe".

### Subjects

Thirty-one healthy and recreationally active subjects participated in this investigation (16 men and 15 women). Subject characteristics were (Mean ± SD), men (n = 16): age, 56.0 ± 6.0 yrs, height, 177.3 ± 3.8 cm, body mass, 85.3 ± 8.9 kg; women (n = 15): age, 52.0 ± 7.5 yrs, height, 168.4.3 ± 4.2 cm, body mass, 75.5 ± 10.7 kg. Subjects were recreationally active (expending approximately 750 to 24,000 METS weekly, with vigorous activity accounting for approximately 2080 ± 2300 METS per week) and participated in various activities (none were trained master athletes).

Our medical monitor screened subjects for pre-existing medical conditions that could put subjects at risk or interfere with the investigation. Exclusion criteria included, but were not limited to: heart conditions or anomalies; respiratory conditions; blood pressure problems; and musculoskeletal problems or previous orthopedic injuries that would limit the range of motion. In addition, participants were excluded if they had recently taken or planned to take any products containing creatine, ephedra, and high doses of caffeine. All subjects were fully informed of the protocol design and associated risks of this investigation before signing an informed consent approved by the University of Connecticut Institutional Review Board for use of human subjects.

#### Procedures

##### Treatment conditions

Subjects were asked to continue their normal workouts during the study and maintain the same level of activity between both cycles. To ensure consistency of their daily activities during the two supplementation periods, subjects were asked to fill out the logbook which included a physical activity report and a medication treatment log for the end of each week. Subjects were asked to fast (other than water as subjects were instructed on hydration protocols with urine specific gravity used for verification) for 12 hours prior to the lab visit. Exercise was not permitted 24 hours prior to visits and all visits occurred in the morning and within a one-hour window of their first visit.

##### Flow Mediated Dilation

Endothelium-dependent FMD was assessed *non-invasively *in the peripheral circulation using high-frequency ultrasonographic imaging (Acuson Corp, Elmwood) Park, NJ) at each testing visit as described [35] with minor modification. Briefly, brachial artery FMD was measured following a 5 min occlusion of the forearm as this method is suggested to better reflect nitric oxide (NO˙)-mediated vascular dilation compared to upper arm occlusion [36]. All vascular measurements and analysis were performed by two trained investigators in a blinded manner. Relative (%) and absolute (mm) FMD were calculated by determining peak post-occlusion vessel diameter relative to pre-occlusion diameter.

##### Biochemical Sampling and Analyses

At each blood draw, 20 ml of blood was collected from the antecubital vein via venipuncture into tubes containing either no preservative (Serum) or EDTA. Tubes were centrifuged at 3000 RPM for 15 min at 4°c. Serum/plasma was transferred into storage tubes and stored at -80°C for future analysis.

As a measure of tissue disruption and recovery status, Creatine Kinase concentration was measured in duplicate using Diagnostics Chemicals Limited (Oxford, CT) reagents. Glucose (for confirmation of fasting) and C-reactive protein (CRP) (a measure of local inflammation) were determined using a Cobas c 111 automated analyzer (Roche Diagnostics, Indianapolis, IN). Glucose and CRP reagents were purchased from Fisher Scientific. IL-6 was determined using the Invitrogen Corporation (Carmarillo, CA) ultrasensitive ELISA. Cortisol was analyzed with the ALPCO Diagnostics (Salem, NH) ELISA to confirm abstinence from exercise and healthy population status. The Immunology Consultants Laboratory, Inc. (Newberg, OR) ELISA was used to determine alpha 1 -antichromotrypsin. All ELISAs were performed on the Molecular Devices VERSAmax tunable microplate reader. Both intra-assay and inter-assay variances were < 5%.

##### Physical Performance Testing

Subjects performed a stationary standing test on a force platform (Advanced Mechanical Technology, Inc., Watertown, MA) to examine balance as determined by dispersion from center of pressure [37]. They were instructed to stand with feet together and arms relaxed and to the side for 40 seconds. A countermovement vertical jump test was also performed on the force platform to determine muscle function and power capabilities [38]. Subjects initiated three maximal effort countermovement vertical jumps on a force plate (with their hands on their waists during the movement). In addition, subjects performed a maximal isometric handgrip strength test [39]. The hand grip was held by their side with their dominate hand. Subjects were asked to squeeze the handgrip instrument as hard and as quickly as possible for approximately 2-4 seconds with a two minute rest period between each repetition. Three trials were performed and the best score was recorded.

##### Perceptual Scales

The Logbook utilized the National Institutes of Health PROMIS-57 Assessment Center scales for functioning [40], the Lequesne Knee Index [41] and the Knee Injury and Osteoarthritis Outcome Score (KOOS)[40,42]. We examined PROMIS-57 subscales of physical function, mood (anxiety), fatigue, and pain. The Lequesne index is a 10-question survey given to individuals with osteoarthritis of the knee accessing pain or discomfort, walking, and activities of daily living. The KOOS consists of 5 subscales: Pain, Symptoms, Function in Daily Living, Function in Sport and Recreation and Knee-Related Quality of life. Perceived energy was adapted from the Multidimensional Fatigue Symptom Inventory-Short Form (MFSI-SF)[43] and reported at the final visit of each cycle using a 5-point Likert scale.

##### Statistical Analyses

The data are presented as means and standard deviations. Area under the curve calculations for blood markers using the trapezoidal method were also calculated for selected variables. Data were evaluated for assumptions for use of linear analysis models before analysis and then corrected with log10 transformations and reanalyzed when appropriate. A two-way analysis of variance was used to examine changes in the distribution. Where significant main effects were determined, an appropriate Tukey's or Fisher's LSD post hoc test were conducted to determine pairwise differences between treatments of sex. Intra-class correlation coefficients were all R ≥ 0.94. The alpha level for significance in this investigatgion was be set at P ≤ 0.05.

## Results

The supplement was well-tolerated by all subjects, both in terms of taste and absence of any side effects. No significant differences were shown between the pre- and post- placebo testing conditions for any variables. In addition, as applicable, no significant differences were seen between placebo and testing variables prior to supplementation.

**Figure 2:**
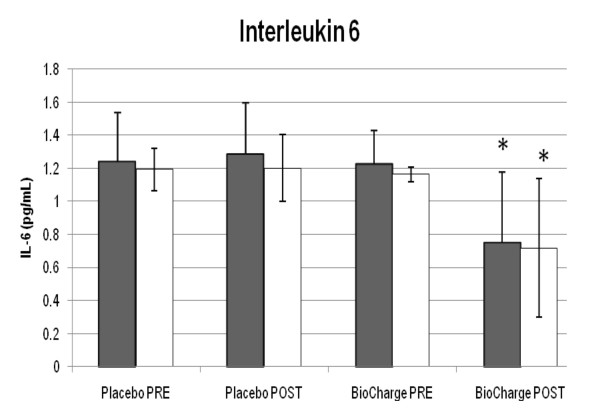
**Interleukin-6 (IL-6)**. There was a significant decrease in interleukin-6 for both men and women from pre-supplementation, * = P ≤ 0.05 from corresponding pre-BioCharge and placebo conditions. Grey: men; white: women.

**Figure 3:**
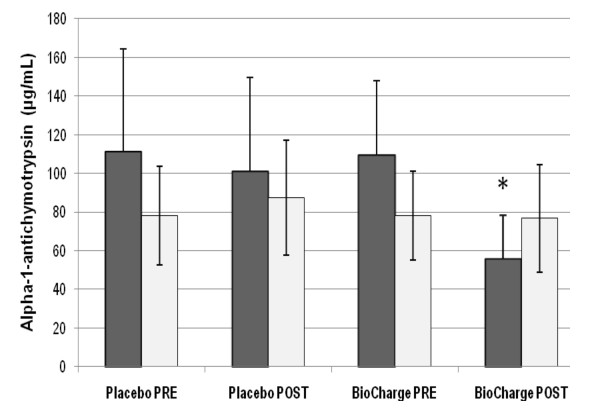
**Alpha-1 Antichymotrypsin**. There was a significant decrease in alpha-1 antichymotrypsin for men from pre-supplementation, * = P ≤ 0.05 from corresponding pre-BioCharge and placebo conditions. Grey: men; white: women.

Men displayed a significant decrease in IL-6 (from 1.2 ± 0.2 to 0.7 ± 0.4 pg·mL^-1^) (Figure 2), Creatine Kinase (from 96 ± 34 to 67 ± 23 IU·L^-1^) (Table 2), and Alpha-1 Antichymotrypsin (from 108.9 ± 38.6 to 55.5 ± 22.2 ug·mL^-1^) (Figure 3), whereas women only showed a significant decrease in IL-6 post supplementation (from 1.16 ± 0.04 to 0.7 ± 0.4 pg·mL^-1^). No differences were seen for cortisol, C- reactive protein, or brachial artery FMD (Table 2).

**Table 2:** Physiological markers of inflammation in response to supplementation

	Men				Women			
**Variables**	**Pre Placebo**	**Post Placebo**	**Pre Suppl**	**Post Suppl**	**Pre Placebo**	**Post Placebo**	**Pre Suppl**	**Post Suppl**

	***M ± SD***	***M ± SD***	***M ± SD***	***M ± SD***	***M ± SD***	***M ± SD***	***M ± SD***	***M ± SD***

Creatine Kinase (IU·L^-1^)	92 ± 25	80 ± 32	96 ± 34	67 ± 23*	68 ± 42	69 ± 50	66 ± 32	62 ± 33

Cortisol (nmol·L^-1^)	557 ± 169	613 ± 128	524 ± 180	515 ± 187	560 ± 149	513 ± 129	518 ± 165	541 ± 148

C-Reactive Protein (mg·dL^-1^)	0.22 ± 0.25	0.19 ± 0.25	0.17 ± 0.24	0.19 ± 0.22	0.10 ± 0.10	0.11 ± 0.09	0.1 ± 0.1	0.12 ± 0.11

Brachial Artery Reactivity (mm)	4.6 ± 0.7	4.8 ± 0.7	4.7 ± 0.8	4.9 ± 0.8	3.7 ± 0.6	4.1 ± 0.6	3.8 ± 0.5	4.1 ± 0.5

No significant differences were seen for placebo (either pre/post placebo or as compared to pre-supplementation). Pre Suppl = Pre-Supplementation testing; Post Suppl = Post-Supplementation testing. *p ≤ 0.05 as compared to supplementation pre-testing.

In terms of the perceptual variables, a significant increase in energy after supplementation was seen for both men (placebo: 1.8 ± 0.7; supplement: 3.7 ± 0.8 AUC) and women (placebo: 1.2 ± 0.7; supplement: 2.8 ± 0.8 AUC) as shown in Table 3. Women also reported a lower anxiety level (from 53.0 ± 8.9 to 45.0 ± 8.3 AUC) that was not seen in men (remained at about 43.0 ± 7.3 AUC). Men showed a significant improvement in fatigue, pain, and joint pain after supplementation, which was not seen in women. No other significant differences were noted among the perceptual subscales.

**Table 3:** Perceptual markers in response to supplementation (area under the curve)

	Men				Women			
**Variables**	**Pre Placebo**	**Post Placebo**	**Pre Suppl**	**Post Suppl**	**Pre Placebo**	**Post Placebo**	**Pre Suppl**	**Post Suppl**

	**M ± SD**	**M ± SD**	**M ± SD**	**M ± SD**	**M ± SD**	**M ± SD**	**M ± SD**	**M ± SD**

Energy	-	1.8 ± 0.7^	-	3.7 ± 0.8*	-	1.2 ± 0.7^	-	2.8 ± 0.8*

Anxiety	43 ± 6.3	42 ± 5.6	43 ± 7.2	43 ± 7.4	50 ± 6.5	49 ± 6.5	53 ± 8.9	45 ± 8.3*

Fatigue	44.9 ± 4.6	43.7 ± 4.5	46.9 ± 5	35.8 ± 6.7*	42.4 ± 9	43.3 ± 5.3	43 ± 9.3	43.2 ± 9.5

Joint Pain	1.1 ± 0.3	1.1 ± 0.3	1.2 ± 0.2	0.5 ± 0.3*	0.7 ± 0.2	0.8 ± 0.1	0.7 ± 0.2	0.7 ± 0.2

Pain Intensity	0.8 ± 0.06	1.1 ± 0.08	1.1 ± 0.09	0.6 ± 0.06*	0.8 ± 0.07	0.7 ± 0.08	0.6 ± 0.06	0.6 ± 0.08

Please note (as applicable) that no differences were seen in placebo (either pre/post placebo or as compared to pre-supplementation). Pre Suppl = Pre-Supplementation testing; Post Suppl = Post-Supplementation testing. *p ≤ 0.05 as compared to supplementation pre-testing; ^p < 0.05 as compared to placebo.

The results of performance testing are seen in Table 4. Men produced significantly higher power during the countermovement jump (from 2642 ± 244 to 3134 ± 282 W) and greater force in the grip strength test (from 42.1 ± 5.9 to 48.5 ± 4.9 kg) after supplementation. Women did not show improved power or strength, but did show a significant improvement in the postural sway test (from 0.52 ± 0.13 to 0.45 ± 0.12 cm).

**Table 4:** Performance responses to supplementation

	Men				Women			
**Variables**	**Pre Placebo**	**Post Placebo**	**Pre Suppl**	**Post Suppl**	**Pre Placebo**	**Post Placebo**	**Pre Suppl**	**Post Suppl**

	**M ± SD**	**M ± SD**	**M ± SD**	**M ± SD**	**M ± SD**	**M ± SD**	**M ± SD**	**M ± SD**

Postural Sway (cm)	0.53 ± 0.10	0.48 ± 0.13	0.51 ± 0.16	0.56 ± 0.22	0.52 ± 0.14	0.52 ± 0.12	0.52 ± 0.13	0.45 ± 0.12*

Vertical Jump (W)	2697 ± 212	2761 ± 322	2642 ± 244	3134 ± 282*	2006 ± 244	2046 ± 282	2077 ± 229	2039 ± 409

Grip Strength (kg)	41.5 ± 6.1	41.8 ± 6.2	42.1 ± 5.9	48.5 ± 4.9*	31.6 ± 4.6	33.3 ± 4.5	33 ± 5.1	33.3 ± 5.5

No significant differences were seen in placebo (either pre/post placebo or as compared to pre-supplementation). Pre Suppl = Pre-Supplementation testing; Post Suppl = Post-Supplementation testing. *p ≤ 0.05 as compared to supplementation pre-testing

## Discussion

The primary finding of the current investigation is that a multi-nutrient supplement improved indices of inflammation and recovery in active, middle-age individuals. In addition to improved energy in both men and women, both sexes displayed sex-specific improvements in performance. This suggests that such a supplement may prolong functionality and physiological performance with age, thereby permitting individuals to continue to practice an active lifestyle.

### Decreased Inflammatory Status with IL-6

There was a decrease in IL-6 for both men and women as shown in Figure 2, indicating a decrease in inflammatory status. The normal cortisol, C-reactive protein levels, and brachial artery reactivity in our study indicated that our population consisted of otherwise healthy individuals unlikely to have localized inflammatory disorders. Whether through inhibition of IL-1β [29], NF-kB [32], or other factors, there may be an association between the plant extracts (cat's claw, green tea extract, quercetin, and taurine) and/or BCAA used in this investigation with the decrease in IL-6. Thus, even in an otherwise healthy population, the supplement effectively reduced inflammatory status in both men and women. While the subjects investigated did not display abnormal starting concentrations of IL-6, this may have important implications, as elevated IL-6 concentrations are associated with mortality, chronic disease, and other conditions [6]. Future research may help to clarify whether elevated normal IL-6 in healthy individuals is related to health outcomes in that population.

### Improved Physical Performance in Men

As shown in Figure 3, alpha-1-antichymotrypsin (ACT) decreased in men but not women; in addition, vertical jump and grip strength also increased in men and not women. Given that men began the study with slightly higher ACT than women, this may suggest that the supplement permitted an increase in strength by reducing inflammation in those with higher levels of ACT.

Joint function may suggest another rationale for the improvement in strength and power performance in men. IL-6 status is associated with joint pain [44] but joint pain did not decrease in women despite a decrease in IL-6. On the other hand, it appears that alpha-1-antichymotrypsin (which did decrease in men but not women) can serve as a more sensitive marker to arthritis than measures such as C-Reactive Protein (which did not change in this investigation) [45]. Thus, whether through decreased inflammation or analgesic effects of BCAA, both overall pain and joint pain (which started slightly higher in men than in women) decreased in men but not in women. Thus the anti-inflammatory nature of the supplement may have contributed to increased performance by reducing joint pain.

While strength increases may have been attributable to reductions in ACT, a decrease in Creatine Kinase (CK) was seen in men and was not seen in women. Once again, men began the investigation with higher CK. The reduced CK has been seen in investigations with taurine supplementation [46] and amino acid supplementation [47] but only to a limited degree with plant extracts such as green tea [48]. At the same time, amino acid supplementation has been shown to improve strength and power performance [49]. Thus, while the anti-inflammatory plant extracts appear to have played an important role in recovery, the amino acids may also have produced improved performance.

Finally, energy improved in both men and women, but fatigue only significantly decreased in men. The decrease in perceived fatigue may have resulted in improved performance. While the increased energy is likely due to the B Vitamins, other ingredients such as quercetin [50] cannot be excluded as contributors to this phenomenon. Thus, the supplementation protocol increased energy in both men and women, but the decrease in fatigue was only seen in men and may have influenced their improved performance on strength and power measures.

### Improved Physical Performance in Women

In the present investigation, women (who have a higher risk of hip fracture than men) displayed improved medial-lateral balance with supplementation. At the same time, women also displayed a decrease in anxiety that was not seen in men (the anxiety level for women at baseline (50th percentile, normalized) was higher than men, but the anxiety level was similar after supplementation (45^th ^percentile for women, 43^rd ^for men)). Postural sway is strongly and linearly related to anxiety [51,52], suggesting that the decreased anxiety may have improved balance in women. Thus, whether through reduced anxiety or otherwise, the multi-nutrient supplementation improved balance in women and thereby reduced a risk factor for falling.

## Conclusions

The purpose of the current investigation was to examine the effects of a multi-nutrient supplement on the recovery free-living, middle aged individuals from their active lifestyles in terms of physical performance, fatigue, mood, and other factors. As shown, men and women responded differentially and saw different benefits from the supplement. Inflammation, as determined by IL-6, clearly showed a benefit in both men and women. Both men and women reported increased energy and men displayed a decrease in perceptual fatigue. Anxiety decreased in women, which may have resulted in the decrease women showed in postural sway. Such an outcome could decrease the risk of hip fracture in women. Men showed a decrease in alpha-1-antichymotrypsin, general and joint pain, and an improvement in physical performance. In all cases, the supplement helped to fight signs of aging and could act to prolong the ability of middle-aged individuals to continue their active lifestyles. It therefore appears that a multi-nutrient supplement is beneficial to recovery, inflammatory status, and performance of active men and women. Given the importance of physical activity itself in counteracting inflammation and other indices of aging, this supplement may promote a cycle of anti-inflammatory activity and anti-aging benefits.

## List of abbreviations

ACT: Alpha-1-antichymotrypsin; BCAA: Branched Chain Amino Acids; CK: Creatine Kinase; CVD: Cardiovascular Disease; FMD: Flow Mediated Dilation; IL-6: Interleukin-6; KOOS: Knee Injury and Osteoarthritis Outcome Score.

## Competing interests

Dr. William J. Kraemer is a member of the Advocare^® ^Sci-Med Board. This investigation was funded by AdvoCare™International, Plano, TX, USA and the company provided all of the supplement and placebos used in the study.

There are no competing interests for the following authors: Courtenay Dunn-Lewis, Brian R Kupchak, Neil A Kelly, Brent A Creighton, Hui-Ying Luk, Kevin D Ballard, Brett A Comstock, Tunde K Szivak, David R Hooper, Craig R Denegar, Jeff S Volek

## Authors' contributions

All authors have reviewed the manuscript.

CDN participated in design, coordinated the study and data collection, interpretation of results, drafting the manuscript. WJK a principal investigator, conceived of the study and its design, and drafting the manuscript. BRK carried out biochemical analyses, drafting the manuscript. NAK participated in the coordination of the investigation. BAC was principally responsible for ultrasound data collection and analysis. HYL participated in the interpretation of data and drafting of the manuscript. KDB contributed to design and data collection for ultrasound measures. BAC participated in the acquisition of the data and write up. TKS participated in the acquisition of the data and write up. DRH participated in the acquisition of the data and write up. CRD assisted in conception and analysis of study, drafting of write up. JSV-a principal investigator conceived of the study, participated in the design of the study and drafting manuscript write up.
